# Targeting Interleukin-7 Signalling in Ulcerative Colitis: Rationale and Emerging Clinical Evidence

**DOI:** 10.3390/life16091572

**Published:** 2026-09-20

**Authors:** Magdalini Manti, Valentina Raspa, Kamal Patel, Sailish Honap

**Affiliations:** 1 Department of Gastroenterology, St Mark’s The National Bowel Hospital, London NW10 7NS, UK; magdalini.manti@nhs.net; 2 Department of Gastroenterology, St George’s University Hospitals NHS Foundation Trust, London SW17 0QT, UK; valentina.raspa@stgeorges.nhs.uk (V.R.); kamal.patel@stgeorges.nhs.uk (K.P.); 3 Infection and Immunity Research Institute, City St George’s, University of London, Cranmer Terrace, London SW17 0RE, UK

**Keywords:** interleukin-7, lusvertikimab, inflammatory bowel disease, ulcerative colitis

## Abstract

Ulcerative colitis (UC) remains difficult to control in a substantial proportion of patients despite an expanding range of advanced therapies. Interleukin-7 (IL-7) is a homeostatic cytokine that supports lymphocyte survival, persistence and intestinal trafficking through signalling via IL-7 receptor-α (IL-7Rα; CD127), providing a rationale for selective pathway inhibition in chronic intestinal inflammation. This narrative review examines the biology of IL-7/IL-7R signalling in inflammatory bowel disease (IBD) and summarises the preclinical, translational and clinical development of lusvertikimab, a humanised monoclonal antibody targeting IL-7Rα. MEDLINE was searched from inception to 31 July 2026, supplemented by reference-list screening, trial registries, conference proceedings and regulatory sources. Experimental studies show that IL-7 supports the persistence of colitogenic effector-memory T cells and contributes to innate immune activation. Human translational data demonstrate enrichment of IL-7R pathway activity in treatment-refractory IBD, association with anti-TNF non-response, and IL-7-mediated upregulation of the gut-homing integrin α4β7. Preclinical IL-7R blockade attenuated experimental colitis, reduced intestinal T-cell trafficking and altered inflammatory responses in UC tissue. In a first-in-human study, lusvertikimab produced sustained receptor occupancy and suppression of IL-7-associated gene expression without broad lymphocyte depletion. In the phase II CoTikiS trial, lusvertikimab improved Modified Mayo Score versus placebo, with significant pooled endoscopic improvement but no significant pooled differences in clinical or endoscopic remission. Early safety findings were reassuring. Selective IL-7Rα blockade therefore represents a biologically distinct therapeutic strategy in UC, although larger controlled studies, biomarker validation and clarification of dose selection and long-term safety are required to define its clinical role and whether combination strategies warrant future evaluation in selected patients.

## 1. Introduction

Ulcerative colitis (UC) is a chronic inflammatory bowel disease characterised by continuous mucosal inflammation of the colon, leading to symptoms such as bloody diarrhoea, urgency, and abdominal pain [1]. It is a relapsing–remitting condition driven by dysregulated immune responses in genetically susceptible individuals [1]. The treatment of UC has changed substantially over the past two decades with the introduction of therapies targeting tumour necrosis factor, leukocyte trafficking, interleukin (IL)-12/23 and IL-23, Janus kinase (JAK) signalling, and sphingosine-1-phosphate receptors [2]. Despite this expanding therapeutic landscape, a substantial proportion of patients do not achieve or maintain remission, and treatment-refractory disease remains an important clinical problem. These limitations have led to continued interest in targeting additional immune pathways that may contribute to the persistence of intestinal inflammation despite existing therapies [2,3].

Interleukin-7 (IL-7) is a homeostatic cytokine with a central role in lymphocyte development, survival and maintenance [4]. Although IL-7 is not considered a classical pro-inflammatory cytokine, its signalling through the IL-7 receptor (IL-7R) supports the survival and persistence of T-cell populations and influences their trafficking to the intestine [5]. These properties are particularly relevant to chronic immune-mediated diseases, in which continued survival and recruitment of activated lymphocytes may contribute to chronic inflammation.

Accumulating evidence from experimental and translational studies has provided a rationale for targeting this pathway in inflammatory bowel disease (IBD). IL-7R signalling is increased in inflamed intestinal tissue, while higher expression of IL7R and associated pathway transcripts has been reported in patients with treatment-refractory disease and in those who subsequently fail anti-TNF therapy [6]. IL-7 can also increase expression of the gut-homing integrin α4β7 on human T cells, providing a link between lymphocyte survival and intestinal trafficking [7]. More recent preclinical data suggest that IL-7 may sustain Th17 responses despite IL-23 blockade, raising the possibility that this pathway contributes to persistent inflammation despite cytokine-directed therapy [6,8].

These observations have led to the clinical development of lusvertikimab (formerly OSE-127), a monoclonal antibody targeting IL-7Rα (CD127) [9]. Early clinical studies have demonstrated sustained receptor occupancy and suppression of IL-7-associated gene expression, while the phase II CoTikiS trial provided evidence of clinical and endoscopic activity in patients with moderately to severely active UC [10,11]. Although these findings remain preliminary, selective IL-7R blockade represents a distinct therapeutic approach that warrants consideration alongside established and emerging treatment strategies in UC.

This narrative review first examines the rationale for targeting the IL-7/IL-7R pathway in ulcerative colitis (UC). Next, the biology of IL-7 signalling and its contribution to intestinal inflammation are outlined, followed by a summary of the preclinical, translational, and clinical evidence for IL-7R blockade. The potential role of selective IL-7R inhibition within the evolving UC treatment landscape is also considered. A structured literature search of MEDLINE via PubMed was conducted from database inception to 31 July 2026. Search terms included combinations of “interleukin-7”, “IL-7”, “IL-7 receptor”, “IL7R”, “CD127”, “ulcerative colitis”, “inflammatory bowel disease”, “lymphocyte trafficking”, “lusvertikimab”, and “OSE-127”. No language restrictions were applied. Reference lists of relevant articles and reviews were also screened to identify additional studies. The MEDLINE search was supplemented by searches of ClinicalTrials.gov, major gastroenterology conference proceedings, regulatory records and relevant manufacturer reports where peer-reviewed data were unavailable.

## 2. Biology of the IL-7/IL-7R Pathway and Implications for IBD

Interleukin-7 (IL-7) is a homeostatic cytokine with an important role in lymphocyte development, survival and maintenance. In contrast to many inflammatory cytokines, IL-7 is produced predominantly by non-haematopoietic cells rather than activated lymphocytes [5]. Within the gastrointestinal tract, human intestinal epithelial cells, including goblet cells, express and secrete IL-7, providing a local source capable of influencing mucosal lymphocyte populations [12]. IL-7 signals through a heterodimeric receptor comprising the IL-7 receptor α-chain (IL-7Rα; CD127) and the common γ-chain (γc; CD132) [5]. CD127 also forms part of the thymic stromal lymphopoietin (TSLP) receptor, together with TSLPR, making preservation of TSLP signalling an important consideration when selectively targeting the IL-7 pathway [13]. Receptor engagement activates the associated JAK1 and JAK3, with subsequent phosphorylation of STAT5. Thereafter, activated STAT5 regulates the expression of genes involved in lymphocyte survival, including members of the BCL2 family [14,15]. In human CD4+ effector and memory T cells, IL-7-mediated activation of the JAK–STAT5 pathway increases BCL2 expression and protects against apoptosis [15].

These functions are important for maintaining the peripheral T-cell pool. IL-7 supports the survival of naïve and memory T cells and promotes homeostatic expansion when lymphocyte numbers fall [16]. Expression of IL-7Rα differs across T-cell populations: conventional CD4+ T cells usually express CD127, whereas naturally occurring FOXP3+ regulatory T cells express much lower levels of the receptor [17]. This differential expression may provide a therapeutic opportunity to inhibit pathogenic effector and memory T-cell populations while relatively preserving regulatory T cells. In chronic immune-mediated disease, however, the same survival signals may also allow activated or memory T cells to persist and sustain inflammation and tissue damage.

FOXP3+ regulatory T cells are CD127-low or CD127-negative, whereas conventional effector and memory T cells generally express higher surface levels of CD127 [17]. Low receptor expression does not mean that Tregs are completely insensitive to IL-7: IL-7 signalling can still occur, but human studies found no IL-7-induced increase in α4 or β7 on Tregs. In inflamed UC explants, IL-7Rα blockade altered effector T-cell programmes and reduced IFN-γ secretion while shifting transcriptional signatures toward relative Treg predominance [6]. These findings support relative preservation of Tregs rather than complete sparing or direct stimulation of Treg growth.

Several observations support the relevance of this pathway to UC. Firstly, genome-wide association studies identified IL7R as one of the candidate genes mapping to UC susceptibility loci [18]. Secondly, experimental studies have shown that mice constitutively overexpressing IL-7 developed chronic colitis together with accumulation of IL-7R-expressing T cells within the intestinal mucosa [19]. Subsequent experiments showed that IL-7 was required not only for the development of colitis but also for its persistence. Colitogenic lamina propria CD4+ effector-memory T cells failed to maintain disease in IL-7-deficient recipients, despite initially proliferating after transfer, and this was accompanied by reduced BCL2 expression [20]. These findings suggested that IL-7 acts primarily as a survival factor for established colitogenic T-cell populations rather than simply as a stimulus for their proliferation. Thirdly, mucosal T cells expressing high levels of IL-7R were capable of transferring chronic colitis in experimental models [21]—later work demonstrated increased IL-7Rα expression on colitogenic lamina propria memory CD4+ T cells and showed that IL-7Rα expression was associated with their capacity to persist and induce chronic intestinal inflammation [22]. Overall, these studies suggest that IL-7 may help sustain mucosal effector-memory T cells that continue to drive intestinal inflammation.

The relevance of IL-7R signalling may extend beyond T cells. IL-7Rα is expressed by several innate lymphoid cell (ILC) populations that contribute to intestinal immune homeostasis and inflammation [5]. In a microbiota-dependent model of colitis arising from loss of T-bet in the innate immune compartment, IL-7Rα-expressing ILCs accumulated within the colonic mucosa and contributed to intestinal inflammation [23]. This work from Powell et al. demonstrated that IL-7R-dependent biology can influence both adaptive and innate components of mucosal immunity and suggests that the contribution of the pathway to intestinal inflammation is unlikely to be explained by T-cell survival alone [23].

Human studies provide the most clinically relevant evidence linking the IL-7/IL-7R pathway to IBD. Belarif and colleagues analysed transcriptomic and cellular datasets comprising up to 500 patients with IBD and 100 controls and found enrichment of IL-7R pathway-related transcripts in inflamed colonic tissue from patients with severe Crohn’s disease and UC who had failed corticosteroids or immunosuppressants, anti-TNF therapy, or anti-α4β7 therapy [6]. In independent UC cohorts, increased pretreatment mucosal expression of IL7R and an associated IL-7R signalling signature was strongly associated with subsequent non-response to anti-TNF therapy [6]. These observations do not establish IL-7R expression as a prospectively validated predictive biomarker but suggest that increased pathway activity may characterise a treatment-refractory inflammatory state.

The reported biomarker performance is promising but not sufficient for clinical use. In the pooled pretreatment analysis, mucosal IL7R expression discriminated primary anti-TNF non-response with an ROC AUC of 0.84, while the 10-gene IL-7R-restricted signature achieved an AUC of 0.93; across four individual cohorts, the 10-gene signature yielded AUCs of 0.883–0.967 [6]. The study did not define a prespecified clinical cutoff or report threshold-specific sensitivity, specificity, calibration, or predictive values, and it evaluated anti-TNF non-response rather than response to CD127. Unlike the vedolizumab UC clinical decision support tool, which was derived in a trial cohort and externally validated in routine practice [24], no comparable IL-7R-based score has been prospectively validated. IL7R should therefore be regarded as a candidate enrichment biomarker, not a treatment-selection test.

IL-7 may also influence the localisation of lymphocytes within the gastrointestinal tract. Human T cells exposed to IL-7 increase surface expression and functional activity of the gut-homing integrin α4β7, thereby increasing their capacity to interact with mucosal addressin cell adhesion molecule-1 (MAdCAM-1) [6,7]. This effect has been demonstrated independently in human T cells and was associated with enhanced intestinal homing [7]. Belarif and colleagues subsequently showed that IL-7 selectively increased α4β7 expression on human effector T cells, whereas other major trafficking receptors were largely unaffected [6]. Notably, this effect was not reproduced in murine T cells, indicating an important species-specific component of IL-7 biology and emphasising the relevance of human translational studies when considering its role in IBD.

The microbiota and epithelial barrier introduce an important uncertainty. In a lymphopenic mouse model, IL-7 regulated intestinal epithelial-cell homeostasis and commensal composition, and inactivation of IL-7R signalling increased susceptibility to chemically induced epithelial injury and colitis [25]. Conversely, IL-7R blockade reduced IL-7Rα-positive ILC accumulation and inflammation in microbiota-driven and Helicobacter-associated experimental colitis [23,26]. No clinical study has yet established how prolonged CD127 treatment affects microbiome composition, permeability or epithelial repair. Longitudinal human studies should therefore include stool metagenomics or metabolomics together with barrier and epithelial-injury measures.

**Figure 1:**
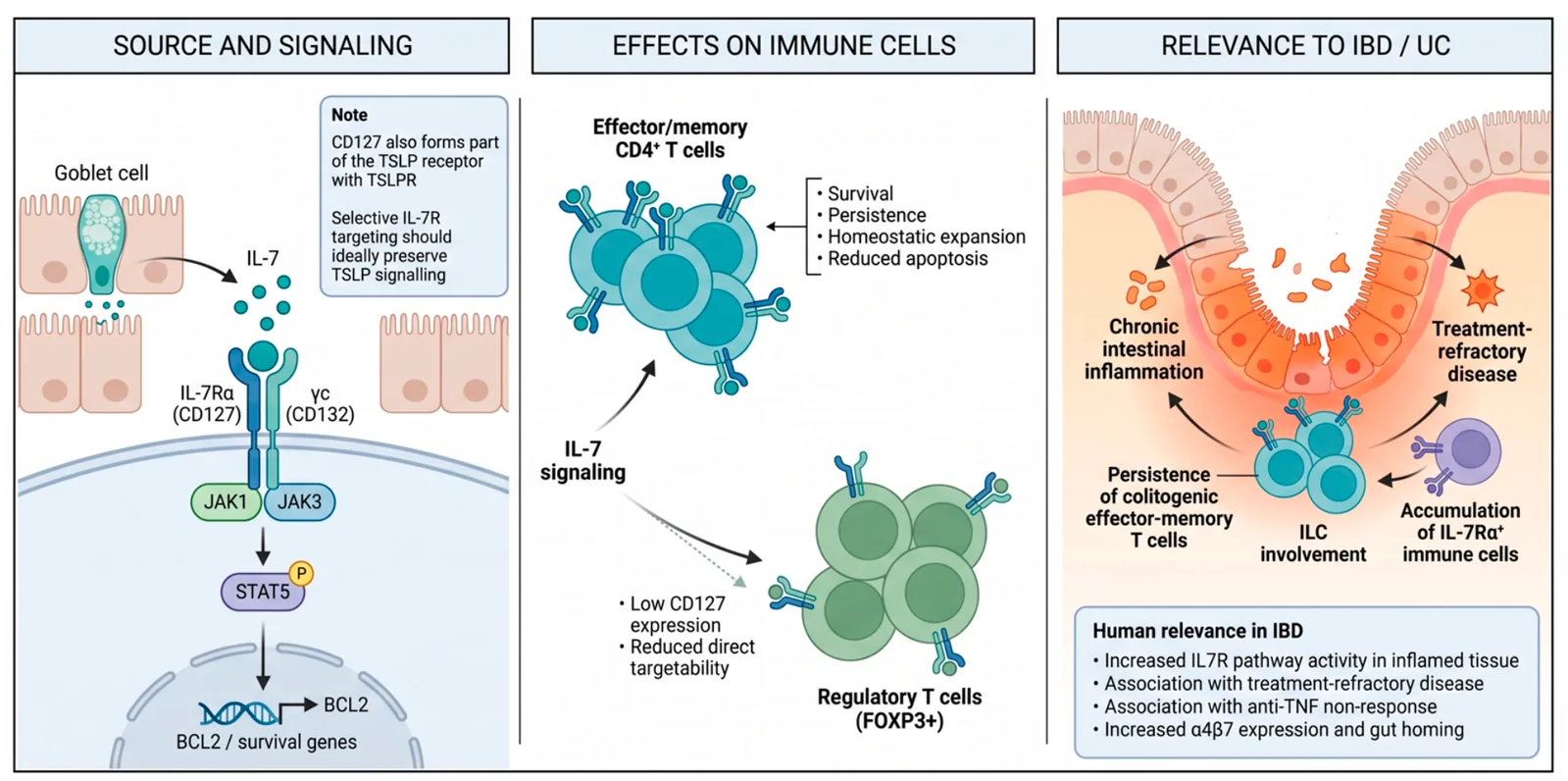
IL-7/IL-7R signalling and its relevance to intestinal inflammation in IBD. IL-7 produced by intestinal epithelial cells signals through IL-7Rα (CD127) and the common γ-chain (CD132), activating JAK1/JAK3–STAT5 signalling and promoting lymphocyte survival. IL-7R signalling may contribute to IBD through persistence of effector-memory T cells, involvement of IL-7Rα-expressing innate lymphoid cells, and enhanced intestinal trafficking, with increased pathway activity observed in treatment-refractory disease. Created in BioRender. Honap, S. (2026) https://BioRender.com/tils7c9.

To summarise, these findings demonstrate that IL-7 contributes to chronic intestinal inflammation largely by sustaining IL-7R-expressing immune-cell populations and, in humans, by facilitating the intestinal trafficking of effector T cells. This differs from several cytokines currently targeted in IBD, whose predominant effects involve the activation or amplification of inflammatory effector responses. Instead, IL-7 may contribute to the persistence of mucosal inflammation by maintaining pathogenic lymphocyte populations and facilitating their continued localisation within the intestine. The enrichment of IL-7R signalling in treatment-refractory human IBD further raises the possibility that this pathway remains active when other inflammatory pathways have been therapeutically suppressed, providing the biological rationale for evaluating selective IL-7R inhibition in UC. Table 1 and Figure 1 summaris the components of the IL-7/IL-7R pathway and their relevance to IBD.

## 3. Preclinical Development of Selective IL-7Rα Blockade

OSE-127 (lusvertikimab) is a humanised IgG4 monoclonal antibody targeting IL-7Rα (CD127) [27]. The antibody was designed as a selective antagonist of IL-7 signalling that does not induce receptor internalisation and was developed to avoid broad cytotoxic depletion of IL-7Rα-expressing lymphocytes [10]. OSE-127 was subsequently assigned the International Nonproprietary Name (INN) lusvertikimab [27,28]. This pharmacological profile provided the basis for evaluating selective IL-7Rα blockade in chronic inflammatory disease and haematological malignancies [29].

Lusvertikimab does not obtain pathway selectivity by binding an IL-7-specific receptor chain given that IL-7Rα is the common subunit of both the IL-7 and the TSLP receptor. However, the selectivity of lusvertikimab is functional and epitope-dependent; it is designed to prevent productive IL-7Rα/common γ-chain signalling while largely sparing TSLPR/IL-7Rα signalling. In the preclinical study preceding the clinical development of lusvertikimab, anti-IL-7Rα IgG4 antibodies inhibited IL-7-induced STAT5 phosphorylation but reduced TSLP-induced CCL17 secretion by dendritic cells by less than 20% [30].

Early evidence that pharmacological IL-7Rα inhibition could improve gut inflammation preceded the development of lusvertikimab. Willis et al. investigated anti-IL-7R treatment in a murine model of *Helicobacter bilis*-induced colitis using both immunocompetent and lymphocyte-deficient mice. IL-7R blockade reduced intestinal inflammation and disease severity in immunocompetent animals, accompanied by effects on several leukocyte populations. Importantly, benefit was also observed in lymphocyte-deficient mice, where treatment reduced the number and activity of macrophages and dendritic cells despite the absence of adaptive lymphocytes [26]. These findings provided early proof-of-concept that IL-7R-dependent inflammation could be therapeutically modified and suggested that the effects of pathway blockade extend beyond T cells to innate immune populations.

Belarif et al. subsequently evaluated selective IL-7Rα antagonism in a non-human primate model of antigen-driven chronic inflammation [30]. This showed anti-IL-7Rα antibodies produced sustained suppression of antigen-specific skin inflammation and reduced IFN-γ-producing memory T-cell responses following repeated antigen challenge. Importantly, these effects occurred without significant changes in peripheral T-cell numbers, phenotype or responses to polyclonal stimulation, supporting the possibility that IL-7Rα blockade can preferentially modulate antigen-experienced memory responses without broad lymphocyte depletion.

The relevance of IL-7Rα inhibition in IBD was later examined by Belarif et al. using complementary humanised mouse models and human UC tissue [6]. Pharmacological IL-7Rα blockade was shown to prevent IL-7-mediated upregulation of α4β7 on human effector T cells and reduced their intestinal trafficking. In humanised mice, anti-IL-7Rα treatment reduced human T-cell infiltration into the colon and attenuated colonic inflammation [6]. In an acute TNBS model, treated mice showed improved weight recovery and less diarrhoea, while in a chronic DSS model, initiation of IL-7Rα blockade after the onset of intestinal inflammation reduced diarrhoea and histological injury [6]. Translational evidence was provided by ex vivo culture of inflamed colonic biopsies from patients with UC, where IL-7Rα blockade reduced IFN-γ secretion and altered effector T-cell transcriptional responses while relatively preserving regulatory T cells. These experiments extended the earlier reported studies by the same group demonstrating activity across human T cells, humanised models of intestinal inflammation and directly studied UC mucosal tissue.

More recent preclinical work has raised the possibility that IL-7R blockade may complement inhibition of the IL-12/23 pathway. In an anti-TNF-refractory adoptive T-cell transfer model of chronic colitis, anti-IL-7R monotherapy had limited activity at the dose studied, while anti-IL-12/23 treatment reduced disease activity without achieving complete macroscopic or histological remission [8]. Combined IL-7R and IL-12/23 blockade produced greater reductions in clinical disease, macroscopic colonic inflammation and histopathological abnormalities than either approach alone, with histological findings reported to be comparable with non-inflamed controls. In parallel, in vitro experiments using human cells, IL-7 supported Th17 differentiation despite IL-23 blockade, whereas combined IL-7R and IL-23 inhibition prevented Th17 generation in the presence of both cytokines. Although these findings are currently available only as a conference abstract and require confirmation in a full peer-reviewed report, they provide preliminary evidence that IL-7R and IL-12/23 signalling may represent complementary pathways in chronic intestinal inflammation.

Preclinical studies support selective IL-7Rα blockade through several observations that include: attenuation of experimental colitis, suppression of antigen-driven memory T-cell responses without broad lymphocyte depletion, reduced trafficking and inflammatory activity of human effector T cells, and activity within human UC mucosal tissue. These findings provided the rationale for progression of lusvertikimab into clinical evaluation.

## 4. Clinical Development of Lusvertikimab

### 4.1. Phase I Study

In 2023, Poirier et al. reported the first-in-human evaluation of OSE-127 in a randomised, double-blind, placebo-controlled, single-centre Phase I study [10]. Sixty-three healthy volunteers were enrolled, of whom 45 received OSE-127 and 18 received placebo. Nine dosing cohorts were assessed, including six single ascending intravenous dose cohorts (0.002–10 mg/kg), one subcutaneous cohort (1 mg/kg), and two cohorts receiving two intravenous doses of 6 or 10 mg/kg [10].

OSE-127 showed dose-dependent pharmacokinetics, with a terminal half-life of up to 11.7 days following single dosing and 16.25 days following repeat dosing. Pharmacodynamic analyses demonstrated suppression of an IL-7-associated six-gene signature, including BCL2, with four genes subsequently showing dose-dependent modulation by quantitative RT-PCR [10]. Treatment was generally well tolerated, with no cytokine-release syndrome and no significant changes in circulating lymphocyte numbers or major lymphocyte subsets. Overall, the study demonstrated sustained target engagement and inhibition of IL-7 signalling without evidence of broad lymphocyte depletion, supporting further clinical evaluation of OSE-127. It also confirmed IL-7 pathway inhibition in humans but did not include a clinical in vivo TSLP pharmacodynamic endpoint [10].

### 4.2. Phase II CoTikiS Study

CoTikiS (NCT04882007) was a multicentre, randomised, double-blind, placebo-controlled phase II induction study in adults with moderately to severely active UC and an inadequate response to conventional therapy and/or failure of advanced therapy [31]. The groups reported in the ECCO 2025 analysis comprised placebo (*n* = 49) and intravenous lusvertikimab 450 mg (*n* = 35) and 850 mg (*n* = 51), administered at weeks 0, 2 and 6. The 450 mg arm was discontinued following a prespecified interim futility analysis, although subsequent analyses showed evidence of efficacy in this dose group [11].

At week 10, lusvertikimab was associated with a greater reduction in Modified Mayo Score than placebo. The placebo-adjusted differences were −1.16 with 450 mg (*p* = 0.019), −0.90 with 850 mg (*p* = 0.036) and −1.00 for the pooled lusvertikimab group (*p* = 0.010) [11]. Clinical remission was achieved in 22.0%, 13.0% and 4.4% of patients receiving 450 mg, 850 mg and placebo, respectively. Endoscopic improvement occurred in 44.6%, 24.3% and 12.6%, while endoscopic remission occurred in 34.7%, 19.4% and 12.6%, respectively. In the pooled lusvertikimab group, endoscopic improvement was significantly more frequent than with placebo (31.9% vs. 12.6%; *p* = 0.027), whereas pooled clinical remission (16.2% vs. 4.4%; *p* = 0.066) and endoscopic remission (25.0% vs. 12.6%; *p* = 0.120) did not reach statistical significance.

Numerically greater efficacy was observed with 450 mg than with 850 mg, although the study was not designed to compare doses directly. Interpretation is limited by early discontinuation of the 450 mg arm, small treatment groups and the exploratory nature of some subsequent analyses. The findings therefore support clinical and endoscopic activity but do not establish a clear dose–response relationship. The apparent advantage of 450 mg should not be interpreted as an inverse dose–response. CoTikiS was not powered for a direct comparison between doses, and the 450 mg arm was stopped early after an interim futility assessment, leaving smaller and unequal groups and increasing sensitivity to chance baseline imbalance and endpoint variability [11]. Both doses met the primary Modified Mayo Score endpoint. Phase I data showed at least 95% receptor occupancy at doses of 0.02 mg/kg or higher, making a pharmacodynamic plateau biologically plausible, but no published exposure-response or tissue-pharmacokinetic analysis has shown that target saturation explains the numerical difference [10].

During the optional 24-week open-label extension, all participants received lusvertikimab 850 mg every four weeks [32]. Data presented at Digestive Disease Week 2025 indicated that 92% of participants who entered symptomatic remission during induction maintained it, while 61% of those who had not initially achieved symptomatic remission subsequently did so [32]. These findings are encouraging but remain difficult to interpret in the absence of a concurrent control group and with reliance on symptom-based outcomes. Table 2 summarises the clinical trial design and key safety and efficacy outcomes.

Treatment was generally well tolerated during CoTikiS, with a safety profile broadly comparable with placebo [11]. Lymphopenia was reported but was predominantly mild and transient and did not lead to treatment interruption or discontinuation or an apparent increase in infections. However, the relatively small number of exposed patients and limited duration of controlled follow-up preclude conclusions regarding less common or longer-term adverse effects.

**Figure 2:**
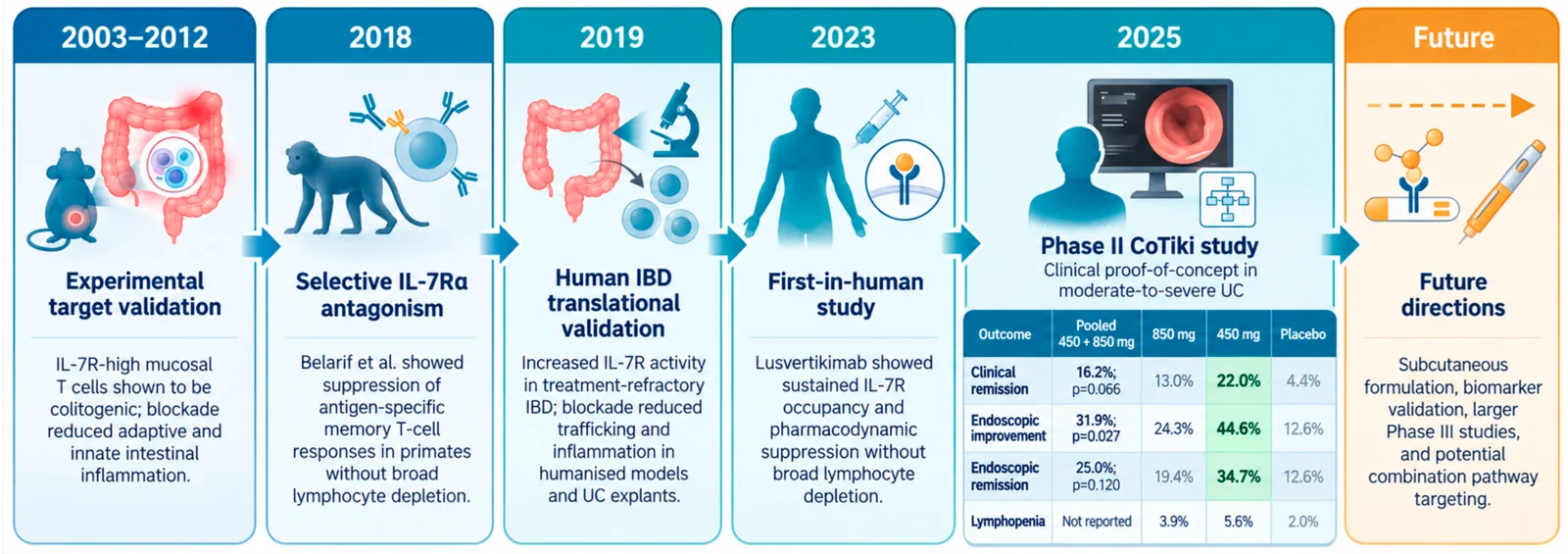
Translational development of selective IL-7Rα blockade in inflammatory bowel disease. Key milestones in the progression of IL-7Rα blockade from experimental target validation and human translational studies to first-in-human evaluation and phase II clinical proof-of-concept with lusvertikimab in UC. Future priorities include subcutaneous development, biomarker validation and confirmation in larger controlled trials. Created in BioRender. Honap, S. (2026) https://BioRender.com/xr0fxp9.

Prolonged blockade still requires dedicated immune-memory surveillance. In primates, antagonist IL-7Rα blockade reduced antigen-specific recall responses while leaving total peripheral T-cell numbers and polyclonal responses largely unchanged; de novo responses could be generated after drug withdrawal [30]. Phase I follow-up was shorter than 146 days, and CoTikiS provided only 10 weeks of placebo-controlled exposure followed by an open-label extension to week 34 [10,11,32]. These data do not exclude uncommon infections, impaired vaccine responses, gradual changes in lymphocyte subsets, or effects on long-lived immune memory. Later trials should monitor absolute counts, major lymphocyte subsets, opportunistic infections and prespecified vaccine or recall-antigen responses over longer follow-up. Figure 2 summarises the translational development of selective IL-7Rα blockade in IBD.

## 5. Conclusions and Future Directions

The IL-7/IL-7R pathway represents a novel therapeutic target in UC, with a rationale that focusses on the survival, persistence and gut trafficking of inflammatory lymphocyte populations. As discussed, experimental and translational studies support a role for IL-7R signalling in chronic and treatment-refractory intestinal inflammation, while the phase II CoTikiS study provides initial clinical proof-of-concept for selective IL-7Rα inhibition. However, at present, the clinical evidence remains limited to a relatively small phase II programme, and the absence of a clear dose–response relationship and controlled maintenance data means that the degree and durability of benefit remain uncertain. Further work is needed to clarify dose selection and establish efficacy across clinical, endoscopic and longer-term outcomes. Longer follow-up will also be important for safety, given the physiological role of IL-7 in lymphocyte homeostasis, although the absence of broad lymphocyte depletion or a clear infection signal to date is reassuring. The future value of IL-7Rα inhibition will also depend on how it compares with an increasingly effective range of advanced therapies for UC.

Patient selection is particularly important. Increased mucosal IL-7R signalling has been associated with treatment-refractory IBD and non-response to anti-TNF therapy, while OSE Immunotherapeutics has reported a potential response-predictive biomarker from CoTikiS that will undergo further evaluation [6,33]. Prospective validation will be required before a biomarker-guided treatment strategy can be considered, but such an approach could help define a clearer therapeutic position for IL-7R blockade.

Drug development pathways in IBD are also evolving with an increasing focus on orally and subcutaneously delivered therapies. OSE Immunotherapeutics is developing a subcutaneous formulation of lusvertikimab, with plans for bioequivalence studies and partnering before further late-phase trial development [33]. At the time of writing, no phase III programme in UC has been initiated according to ClinicalTrials.gov. Beyond UC, chronic pouchitis and hidradenitis suppurativa have been selected for further clinical development [34]. Lusvertikimab also received EU orphan medicinal product designation for acute lymphoblastic leukaemia in July 2023 [35]. Conversely, a randomised phase IIa study in primary Sjögren’s syndrome showed no difference from placebo in mean ESSDAI change at week 13, highlighting that the importance of IL-7R signalling may vary between immune-mediated diseases [36].

Lusvertikimab remains investigational; hence, the marketed price or formal cost-effectiveness estimate is unavailable. The CoTikiS regimen required intravenous infusions at weeks 0, 2 and 6 and, in the extension, every four weeks, creating administration and travel burdens that should be compared with oral, subcutaneous and less frequent maintenance options [11]. A subcutaneous formulation is planned but has not established clinical equivalence or an implementation cost [30]. Published CoTikiS reports have not provided validated quality-of-life, work-productivity or health-resource outcomes. Later-phase studies should prospectively collect validated IBD-specific quality-of-life measures alongside data on work productivity, hospitalisation and surgery, infusion-resource utilisation, and direct and indirect costs to enable a robust assessment of comparative value [37].

An additional question is whether IL-7R blockade will ultimately be most effective as monotherapy or through complementary pathway inhibition. As discussed above, preclinical evidence that IL-7 can sustain Th17 responses despite IL-23 blockade, together with greater suppression of experimental colitis following combined IL-7R and IL-12/23 inhibition, provides a rationale for further investigation. These findings remain preliminary and would require confirmation before clinical combination strategies are considered.

Overall, selective IL-7Rα blockade introduces a different approach to immune modulation in UC by targeting processes that may contribute to the persistence rather than simply the amplification of intestinal inflammation. Lusvertikimab has provided early evidence that this biology can translate into therapeutic activity, but its future role will depend on confirmation in larger controlled studies, validation of predictive biomarkers and clearer positioning within the expanding UC treatment landscape.

## Figures and Tables

**Table 1:** Summarises the principal roles of the IL-7/IL-7R pathway relevant to IBD.

Biological Feature	Role of IL-7/IL-7R Signalling	Evidence Relevant to IBD	Potential Therapeutic Relevance
**T-cell survival and persistence**	Promotes survival of naïve and memory T cells through JAK1/JAK3–STAT5 signalling and induction of anti-apoptotic proteins including BCL2	IL-7 is required for the persistence of experimental colitis; IL-7Rα-high mucosal and lamina propria memory CD4+ T cells are associated with colitogenic activity	IL-7R blockade may reduce the persistence of pathogenic effector-memory T-cell populations
**Innate immune responses**	IL-7Rα is expressed by subsets of innate lymphoid cells involved in mucosal immunity	IL-7Rα-expressing ILCs accumulate in experimental colitis and contribute to intestinal inflammation	IL-7R inhibition may influence both adaptive and innate components of intestinal inflammation
**Treatment-refractory inflammation**	Increased IL-7R pathway activity may identify sustained lymphocyte survival signalling within inflamed mucosa	IL-7R pathway transcripts are enriched in treatment-refractory IBD, and increased pretreatment mucosal *IL7R* expression is associated with anti-TNF non-response	Potential therapeutic target and candidate biomarker of treatment refractoriness
**Intestinal trafficking**	IL-7 increases expression and activity of the gut-homing integrin α4β7 on human T cells	IL-7 enhances α4β7-mediated interaction with MAdCAM-1 and promotes intestinal homing of human effector T cells	IL-7R inhibition may reduce recruitment of inflammatory T cells to the intestinal mucosa
**Selective pathway inhibition**	IL-7Rα provides a receptor-level target upstream of JAK1/JAK3–STAT5 signalling	Pharmacological IL-7Rα blockade reduces intestinal inflammation and T-cell trafficking in experimental and translational models	Provides a more selective approach to IL-7 signalling than inhibition of shared downstream JAK pathways

**Table 2:** Clinical development of lusvertikimab in ulcerative colitis.

Study	Population and Treatment	Key Clinical Message	Main Limitation
Poirier et al., 2023 [10]; Phase I	63 healthy volunteers; single and repeated IV dosing and one SC cohort	Sustained IL-7R occupancy and pharmacodynamic suppression of IL-7 signalling without broad lymphocyte depletion	Healthy volunteers; no efficacy assessment
CoTikiS induction; Phase II	Moderately to severely active UC; placebo or lusvertikimab 450 or 850 mg IV at weeks 0, 2 and 6	Evidence of clinical and endoscopic activity; pooled endoscopic improvement was significant, whereas pooled clinical and endoscopic remission were not	Small and unequal groups; 450 mg arm stopped early; unclear dose–response; short controlled period
CoTikiS open-label extension	Optional extension; lusvertikimab 850 mg every 4 weeks to week 34	Symptomatic remission was maintained in most induction responders, with additional patients achieving remission during continued treatment	No concurrent control; optional entry; symptom-based outcomes

## Data Availability

No new data were generated or analysed for this narrative review. All information discussed was obtained from the publicly available sources cited in the article.

## Abbreviations

The following abbreviations are used in this manuscript:

IBD	Inflammatory bowel disease
IL-7	Interleukin 7
IL-7R	IL-7 receptor
INN	International Nonproprietary Name
UC	Ulcerative colitis
UCEIS	Ulcerative Colitis Endoscopic Index of Severity
